# Society Meetings

**Published:** 1899-02

**Authors:** 


### SOCIETY MEETINGS.

The Medical Union at its annual meeting, which was held Wednesday night, December 28, 1898, at the Genesee Hotel, elected the following-named officers: President, Dr. J. C. Thompson; vice-president, Dr. Herman Mynter; secretary and treasurer, Dr. Frederick Preiss; executive committee, Dr. A. H. Briggs, Dr. A. A. Hubbell and Dr. Frederick Preiss.

---

The Niagara Falls Academy of Medicine and the Niagara Frontier Dental Society held a joint meeting at the Tower Hotel, Monday evening, January 9, 1899. A paper was read by Dr. D. F. Bentley, subject, Diseases of the antrum, which was discussed by Drs. O. E. McCarty and F. T. Jenkins. A paper was read by Dr. C. G. Leowolf, subject, Operation on teeth during pregnancy, which was discussed by Drs. G. J. Musgrove and P. E. Redpath. Following the papers and discussion, a banquet was served.

---

The Sixth International Otological Congress will be held in London, from August 8 to August 12, 1899, under the presidency of Dr.

Urban Pritchard. The following subject is down for general discussion: Indications for opening the mastoid in otitis media suppurativa chronica, but papers are desired on any subject relating to otology. The secretary of the executive committee is E. Cresswell Baber, No. 46 Brunswick square, Brighton, to whom communications may be addressed. This announcement recalls the fact that the first of these congresses was held in Chickering Hall, New York, in October, 1876, under the presidency of Dr. D. B. St. John Roosa.

---

The Surgical Section of the Buffalo Academy of Medicine held its first meeting of the year, January 3, 1899, and was called to order by Dr. J. Henry Dowd, as chairman, *pro tem.* A very interesting paper, Protargol in acute gonorrhea, was presented by Dr. George Knowles Swinburn, Surgeon Good Samaritan Hospital, instructor in genito-urinary diseases Cornell Medical College and editor *Journal of Genito-urinary and Cutaneous Diseases*, New York. Discussion by Drs. Howell, Hartwig, C. P. Smith, Wende and Russell. The second paper of the evening was presented by Dr. A. E. Diehl, on Burns, scalds and frost bites. Discussion by Drs. Swinburn, Wende, Thornbury, C. P. Smith, Frederick, Eugene Smith, Denton and Lustig. Dr. Patterson, of the State Hospital, showed a most interesting specimen of rupture of a ventricle. On motion of Dr. Ring, the thanks of the Academy were tendered Dr. Swinburn for coming to Buffalo and personally giving his experience with the drug protargol.

---

The Esculapian Club will hold its February meeting at the residence of Dr. Clark E. Ernest, 282 W. Ferry street, at which time Dr. C. J. Carr will be the essayist.

---

The American Electro-therapeutic Association held its eighth annual meeting at Buffalo, in the rooms of the Society of the Natural Sciences, September 13 to 15, 1898, under the presidency of Dr. C. R. Dickson, of Toronto. The Mayor of Buffalo, Dr. Conrad Diehl, delivered an address of welcome, to which Dr. F. B. Bishop, of Washington, D. C., responded.

The officers for the following year are: President, Dr. F. B. Bishop, Washington, D. C.; first vice-president, Dr. Ernest Wende, Buffalo, N. Y.; second vice-president, Dr. W. H. White, Boston, Mass.; secretary, Dr. D. J. Gerin, Auburn, N. Y.; treasurer, Dr.

R. J. Nunn, Savannah, Ga. Executive council—for three years, Drs. R. Newman, New York, and G. B. Massey, Philadelphia, Pa.; for two years, Drs. A. D. Rockwell and Wm. J. Morton, New York; for one year, Drs. C. R. Dickson, Toronto, Ont., and F. Schavoir, Stamford, Conn. The next meeting will be at Washington, D. C., September 19 to 21, 1899.

A resolution was passed urging colleges and medical schools to establish chairs on electro-therapeutics, or devote more time and attention to teaching this branch, and it was decided to call the attention of the Association of Medical Colleges to the necessity for such a step. The University of Buffalo was congratulated upon having a chair of electro-therapeutics in its medical department. An excellent exhibition of electrical apparatus was held in the lecture-hall next the room of meeting, and proved a very attractive feature.

---

THE Buffalo Academy of Medicine held meetings during the month of January, 1899, as follows:

*Section on Medicine.*—Tuesday evening, January 10th, program: Auto-intoxication, A. M. Hurd; Membraneous enteritis as found in nervous diseases and insanity, H. A. Wood; The relation between physical and mental diseases, H. P. Frost; A few notes on paresis, Helene Kuhlman; Notes of cases, C. J. Patterson.

*Section on Pathology.*—Tuesday evening, January 17th, program: The Pathology of catarrhal deafness, F. W. Hinkel; discussion opened by H. Y. Grant.

*Section on Obstetrics.*—The regular meeting of this section, which should have occurred on Tuesday evening, January 24th, was postponed until Tuesday evening, January 31, 1899, owing to the absence in Europe of Dr. William P. Spratling, essayist.

